# Barriers and Facilitators of Sleep Restriction Therapy in Internet‐Delivered CBT‐I: A Qualitative Content Analysis and the Development of a Treatment Path Model

**DOI:** 10.1111/jsr.70018

**Published:** 2025-02-24

**Authors:** Laura Simon, Lisa Steinmetz, Nils Berghoff, Charlotte Rehm, Lena‐Marie Neumann, Ann‐Marie Küchler, Dieter Riemann, David Daniel Ebert, Kai Spiegelhalder, Harald Baumeister

**Affiliations:** ^1^ Institute of Psychology and Education, Department of Clinical Psychology and Psychotherapy Ulm University Ulm Germany; ^2^ Department of Psychiatry and Psychotherapy, Faculty of Medicine, Medical Center University of Freiburg Freiburg Germany; ^3^ GET.ON Institut für Online Gesundheitstrainings GmbH (Operating Under the Registered Brand ‘HelloBetter’) Hamburg Germany; ^4^ Department of Sport and Health Sciences Technical University of Munich Munich Germany

**Keywords:** adherence, digital health, insomnia, sleep restriction therapy

## Abstract

Sleep Restriction Therapy (SRT) is a core component of cognitive behavioural therapy for insomnia (CBT‐I). Yet, adherence to SRT can be a major challenge for patients. As this challenge is evident in conventional face‐to‐face therapy, it raises the question of adherence to SRT in internet‐delivered therapy. This qualitative study investigates adherence to SRT in internet‐delivered CBT‐I and aims to identify candidate variables that can guide treatment decisions. Participants diagnosed with insomnia who participated in an internet‐delivered CBT‐I as part of a stepped‐care model were interviewed. Data from interviews with 23 participants were included in this study. A qualitative content analysis was employed. Hindering and facilitating individual, intervention‐related, and e‐coaching‐related factors were examined. Moreover, the consequences of SRT (e.g., negative effects and attrition) were examined. Eleven participants consistently implemented SRT, two rejected it from the beginning, eight discontinued, and two showed inconsistent adherence. Docility, low levels of frustration tolerance, and previous negative experiences with SRT emerged as hindering individual factors. In contrast, a high level of insomnia‐related burden, social support, perceived individual fit with the program, and being satisfied with the therapeutic guidance appeared to foster adherence. Based on the results of this study, a treatment path model is introduced, postulating candidate predictors for the indication for SRT within internet‐delivered CBT‐I (e.g., docility and previous negative experiences with SRT), candidate predictors for engagement (e.g., level of commitment and social support), and potential variables for process monitoring (e.g., negative effects and satisfaction with and need for therapeutic guidance).

## 1 Introduction

Cognitive behavioural therapy for insomnia (CBT‐I) is recommended as the first‐line treatment for insomnia disorder (Qaseem et al. 2016; Riemann et al. 2017, 2023). While the influence of adherence on the efficacy of CBT‐I has not been shown unequivocally in empirical studies, it is presumed that therapy adherence is probably a critical factor for the success of CBT‐I (Clough and Casey 2011; Mellor et al. 2022). However, adherence, here defined as patients consistently following the prescribed treatment recommendations (Mellor et al. 2022; Steinmetz et al. 2023), appears to be a challenge in conventional face‐to‐face therapy (Koffel et al. 2017). A recent systematic review identified consistent evidence for the following factors as predictors of adherence to CBT‐I: improvements in sleep after a session, lower levels of dysfunctional beliefs about sleep, greater psychosocial support, and greater self‐efficacy, whereas no consistent evidence was found for demographic variables such as age or gender, mental health symptoms, and baseline insomnia severity (Mellor et al. 2022).

One of the core treatment components of CBT‐I is sleep restriction therapy (SRT), which involves temporally restricting the time in bed (minimum bedtime: 5 h) to match the average self‐reported sleep duration (Spielman et al. 1987). Adherence to SRT can be particularly challenging, as it involves considerable behavioural and lifestyle changes. However, evidence exists that SRT may be an important driver of the efficacy of CBT‐I (Furukawa et al. 2024; Maurer et al. 2020). Besides the changes in the daily structure, SRT can also lead to negative effects, such as mood changes, fatigue/exhaustion, sleepiness, and irritability (Kyle et al. 2011), which may also impact adherence to SRT. In this regard, researchers have emphasised the need for studies to determine whether negative effects contribute to attrition in CBT‐I (Crawford et al. 2016; Luik et al. 2019).

Given the existing treatment gap due to the limited availability of therapists offering CBT‐I and the high prevalence of insomnia (Roach et al. 2021), various internet‐delivered CBT‐I programs (iCBT‐I) have been developed and investigated. However, considering that adherence is already challenging in conventional face‐to‐face CBT‐I (Vincent et al. 2008), it is crucial to explore whether and how adherence to SRT can be achieved in iCBT‐I. Differences in adherence between iCBT‐I and conventional face‐to‐face CBT‐I may exist, as patients receive limited or no therapeutic guidance in iCBT‐I. Yet, the support of a therapist might be important to address doubts or problems encountered during SRT.

Understanding the factors that facilitate or hinder adherence to SRT may not only be used to foster adherence to SRT but could inform research on patients' characteristics that should be considered when determining their general suitability for iCBT‐I. Moreover, this research may help identify candidate predictors that could guide the individualisation of iCBT‐I.

To thoroughly explore the factors that hinder or facilitate adherence to SRT, this study aims to qualitatively investigate the subjective experiences of individuals participating in iCBT‐I with varying degrees of therapeutic guidance. In particular, the following research questions will be explored:

1. Do participants consistently adhere to SRT?
2. Which individual factors hinder and foster adherence to SRT?
3. Which intervention factors hinder and foster adherence to SRT?
4. How does guidance through an e‐coach influence adherence to SRT?
5. What are the consequences of the implementation of SRT (e.g., negative effects, positive effects, and influence on attrition)?

## 2 Methods

We present a sub‐study of the qualitative evaluation of GET Sleep (Spiegelhalder et al. 2022). The current study is reported following the Standards for Reporting Qualitative Research (SRQR; O'Brien et al. 2014); the SRQR‐Checklist is presented in Supporting Information 1.

### 2.1 GET Sleep: Intervention and Cluster Randomised Trial

GET Sleep is a cluster randomised trial investigating three versions of a stepped‐care model for the treatment of insomnia (Spiegelhalder et al. 2022). All participants had been diagnosed with insomnia by their general care physician applying the ICD‐10 criteria (World Health Organization 1992). Detailed information regarding the inclusion and exclusion criteria of GET Sleep can be found in (Spiegelhalder et al. 2022). In the first step, patients were offered psychoeducational treatment from a general care physician. If participants chose to continue, they progressed to the second step of the stepped‐care model, an iCBT‐I. The iCBT‐I included the treatment components psychoeducation, SRT, stimulus control therapy, relaxation therapy, and cognitive therapy targeting rumination and worry. The program was structured into eight sessions (approximately 45–60 min each), and the suggested session frequency of the iCBT‐I was one session per week. In the second session, participants were introduced to SRT. The recommended time in bed (TIB) was automatically calculated based on sleep diary data that patients entered as part of the intervention. The following algorithm was used to calculate the total time in TIB: During the first week of SRT, the TIB was set based on the average total sleep time reported by participants in their sleep diaries. In subsequent weeks, if sleep efficiency (SE), which is the ratio between the total sleep time and the TIB, was below 80%, the TIB was reduced by 30 min, with a minimum TIB of 5 h. The TIB remained unchanged if SE was between 80% and 90%. If SE exceeded 90%, participants had the option to either maintain their current TIB or extend it by 30 min. Participants were able to adjust their sleep window according to their preferred bedtimes and wake times using the iCBT‐I platform. The second session also provided information on potential negative effects and coping strategies. The third session revisited key elements of SRT.

GET Sleep investigated three intervention groups (IG) that differed in the intensity of the therapeutic guidance: All participants had an initial call before the start and a final call upon completion of the iCBT‐I with an e‐coach (i.e., trained and supervised psychologists). In the standard condition (IG1), the participants received written, semi‐standardised feedback from their e‐coach via the messaging function of the intervention platform after each session. In the guidance‐on‐demand condition (IG2), participants had the option to reach out to their e‐coach when needed, but their e‐coach did not actively contact them. In the basic condition (IG3), there was no contact between the participants and the e‐coach except for the initial and final call. After the iCBT‐I, the patients had the option to progress to a specialised medical face‐to‐face treatment in step three of the stepped care model. For more information on the trial, see Spiegelhalder et al. (2022). For the evaluation of GET Sleep, quantitative data was assessed at baseline, 4 weeks after baseline, 12 weeks after baseline, and 6 months after baseline. Data from the baseline survey will be used to characterise the sample. In particular, the Insomnia Severity Index (Bastien et al. 2001) and the Pittsburgh Sleep Quality Index (Buysse et al. 1989) are reported to quantify the sleep‐related symptoms of the participants. The baseline sleep efficiency was calculated using the data from the Pittsburgh Sleep Quality Index. Moreover, depressive symptoms are reported using the Quick Inventory of Depressive Symptomatology—Self Report (Rush et al. 2003), anxiety symptoms using the Generalised Anxiety Disorder 7 questionnaire (Spitzer et al. 2006) and somatic symptoms using the Somatic Symptom Scale‐8 (Gierk et al. 2014). Additionally, information on the use of over‐the‐counter and prescription sleep medication at baseline is reported. Furthermore, usage data of the iCBT‐I was assessed.

### 2.2 Sampling Strategy

The study at hand is a sub‐study of a larger qualitative investigation of GET Sleep focusing on (a) the perceived effectiveness of the stepped‐care model, (b) the treatment process, (c) the online platform, (d) the therapeutic relationship, (e) negative effects and (f) reasons for dropout. The core aim of the larger qualitative investigation was to assess barriers and facilitators of the entire stepped‐care model. As the aim of the current study was to identify factors related to adherence in SRT, particularly the themes of (c) the online platform, (d) the therapeutic relationship, (e) negative effects and (f) reasons for dropout were of interest. Participants of GET Sleep who consented to be contacted for further studies were eligible for the qualitative main study. Treatment response and the completion of the iCBT‐I program were utilised for a purposeful sampling strategy, as they can be seen as key indicators of successful treatment. Treatment response was determined based on the Insomnia Severity Index (Bastien et al. 2001) from baseline to 6 months after baseline, with a reduction of 8 points or more classified as a treatment responders and smaller reductions or increases considered treatment non‐responders (Morin et al. 2011). Based on the platform usage data, iCBT‐I completers were defined as participants who completed at least 80% of the iCBT‐I within 12 weeks, while iCBT‐I dropouts completed less than 80% within the same timeframe. The recruitment aimed to achieve the following distribution of treatment response and iCBT‐I completion: 2/3 treatment responders and 1/3 treatment non‐responders, and 2/3 iCBT‐I completers and 1/3 iCBT‐I dropouts. These proportions were based on the anticipated distributions for iCBT‐I completion and treatment response in the GET Sleep sample. In addition, secondary recruitment considerations included type of IG, age, and gender: To investigate the impact of the intensity of therapeutic guidance, it was intended to recruit approximately equal numbers of individuals from the three IGs. As experiences with the stepped‐care model may differ by gender and age, an equal representation of males and females and individuals both under and over 50 years old was sought. Ethical approval for participation in the main and qualitative studies was obtained from the Ethics Committee of the Medical Center—University of Freiburg. Informed consent was obtained from all participants before their inclusion in the qualitative sub‐study. Participants received 50€ for participating in the qualitative study.

### 2.3 Interview Guide

An interview guide was developed to guide the semi‐structured interviews. As other aspects were investigated in the larger qualitative investigation, only statements pertaining to the research questions presented above were analysed for the current study. The process of formulating the questions for the interview guide followed the principles outlined by Helfferich (2011). Main themes were explored, starting with a broadly formulated guidance question designed to be as open‐ended and narrative‐generating as possible. Moreover, additional prompts were listed in the interview guide if interviewees did not address the respective aspects while answering the guidance questions (Helfferich 2011). Interviewers were encouraged to ask spontaneous follow‐up questions if needed for clarification or to explore unexpected facets. A translated version of the interview guide can be found in Supporting Information 2. The interview guide was developed with an intended duration of 45–60 min.

### 2.4 Data Collection

Interviews were conducted between June and July 2022. Graduate psychology students (author NB as well as VK and DS, see Acknowledgments) interviewed participants. Before conducting the interviews, the interviewers were trained in mock interviews. Interviews were conducted online via Zoom (Zoom Video Communications Inc. 2022) to provide convenience and flexibility to participants (Archibald et al. 2019). For data protection purposes, interviews were conducted with video disabled, and only audio data was recorded using the Zoom recording function. Subsequently, the recordings were transcribed by an external transcription service *abtipper.de*, using simple transcription guidelines (i.e., transcribed verbatim and essentially written down as spoken (Claussen et al. 2020)). Grammatical errors and filler words were retained, but dialect variations were adjusted to standard German.

### 2.5 Qualitative Content Analysis

To explore facilitating and hindering factors for adherence to SRT, a qualitative approach utilising a qualitative content analysis (Kuckartz and Rädiker 2022) was chosen. This method was selected for its ability to construct categories both deductively and inductively. The working group comprised psychologists with diverse backgrounds: one clinical psychologist specialising in digitalisation and insomnia (LSi), another focusing on conventional face‐to‐face therapy for insomnia (LSt), and five individuals new to insomnia therapy, including two graduate students (VK, DS, and NC) and two psychology research assistants (LN and CR). No members of the research team had personal connections with any of the participants.

MAXQDA (Version 20.4.2; (VERBI Software 2021)), a software for qualitative data analysis, was used to conduct the analyses. The main categories were deductively constructed based on the literature on facilitating and hindering factors of CBT‐I adherence (Mellor et al. 2022) and the interview guide. The main categories included the following categories: adherence to SRT, individual factors, intervention factors, e‐coaching and effects. The interviews were coded using these main categories. Segments irrelevant to the research questions were not coded. Text segments addressing multiple categories were allowed to be assigned to multiple categories. Next, text segments from the main categories were considered to derive the sub‐categories inductively. A second round of coding was carried out, in which the previously coded text segments were assigned to sub‐categories, which are presented in detail with the related main categories in Supporting Information 3.

To ensure reliability, two individuals independently coded each interview for both main and sub‐categories. The coders—LSi, LSt, LN, CR and NB—were paired in different combinations for the coding tasks. Coding teams were regularly rotated to incorporate diverse perspectives. All codings were compared, and discrepancies were resolved by discussion. Based on the text segments on adherence to SRT, participants were categorised into rejecting SRT from the beginning on (rejected), discontinuing SRT prematurely (discontinued), inconsistently adhering to SRT (inconsistent), and consistently adhering to SRT (consistent). In the last step, a category‐based evaluation was conducted. Hereby, participants were differentiated by their IGs (i.e., standard, guidance‐on‐demand, basic) and adherence to SRT (i.e., rejected, discontinued, inconsistent, and consistent). For the quotes presented in the results section, individual acronyms were created for each participant, indicating the IG, gender, age in decade, and adherence to SRT.

## 3 Results

Fifty‐five participants from the IGs were invited to partake in interviews. Of these, 26 participants agreed to participate, five actively declined, and the remaining individuals did not respond to the invitations. One participant withdrew from the overall study after the interview, while two participants did not proceed to the iCBT‐I (i.e., the second step of the stepped‐care model). Consequently, data from interviews with 23 participants were available for this sub‐study. Table 1 summarises the characteristics of the interviewed participants. Based on the Insomnia Severity Index (Bastien et al. 2001), 30.4% of participants exhibited subthreshold insomnia severity, while 52.2% reported moderate symptom severity. Baseline data on depressive symptoms, anxiety symptoms, and somatic symptoms suggest a mild level of comorbid symptomatology. Supporting Information 4 provides a detailed description of the participants. The actual ratios of age and iCBT‐I completion closely matched the targeted ratios. However, the targeted ratios for treatment response, gender and IGs were not achieved.

**TABLE 1:** Participant characteristics (*N* = 23).

	*n* (%) or mean (SD)
Gender	
Male	6 (26.1%)
Female	17 (73.9%)
Age (in years)	
20–29	4 (17.4%)
30–39	4 (17.4%)
40–49	2 (8.7%)
50–59	8 (32.8%)
60+	5 (21.7%)
Insomnia Severity Index (ISI)	17.8 (3.6)
Subthreshold (ISI: 8–14)	7 (30.4%)
Moderate (ISI: 15–21)	12 (52.2%)
Severe (ISI: 22–38)	4 (17.4%)
Pittsburgh Sleep Quality Index	11.8 (2.8)
Sleep efficiency	67.5 (14.9)
Depressive symptoms (QIDS‐SR‐16)	8.7 (3.8)
Anxiety symptoms (GAD‐7)	6.8 (3.5)
Somatic symptoms (SSS‐8)	10.9 (4.0)
Usage of over‐the‐counter sleep medicine	
Never	11 (47.8%)
Seldom (less than twice a month)	3 (13.0%)
Regularly (once to twice a week)	4 (17.4%)
Almost every night	2 (8.7%)
Occasionally, if needed (e.g., stressful days; before important days)	3 (13.0%)
Usage of prescription sleep medicine	
Never	16 (69.6%)
Seldom (less than twice a month)	0 (0%)
Regularly (once to twice a week)	1 (4.3%)
Almost every night	3 (13.0%)
Occasionally, if needed (e.g., stressful days; before important days)	3 (13.0%)
iCBT‐I completion	
Completer	17 (73.9%)
Dropout	6 (26.1%)
Treatment response	
Responder	10 (43.5%)
Non‐Responder	13 (56.5%)
Intervention group	
IG1 (Standard condition)	13 (56.5%)
IG2 (Guidance‐on‐demand)	6 (26.1%)
IG3 (Basic condition	4 (17.4%)

*Note*: Insomnia severity was assessed using the Insomnia Severity Index (ISI; Bastien et al. 2001). Sleep‐related symptoms were assessed using the Pittsburgh Sleep Quality Index (Buysse et al. 1989). Sleep efficiency was calculated using the items of the Pittsburgh Sleep Quality Index. Depressive symptoms were assessed via the Quick Inventory of Depressive Symptomatology—Self Report (Rush et al. 2003), anxiety symptoms via the Generalised Anxiety Disorder‐7 questionnaire (Spitzer et al. 2006), and somatic symptoms via the Somatic Symptom Scale‐8 (Gierk et al. 2014). Completers were defined as participants who completed at least 80% of the iCBT‐I within 12 weeks, while dropouts completed less than 80% within the same timeframe. Treatment response was determined based on the Insomnia Severity Index score from baseline to 6 months after study inclusion, with a reduction of 8 points or more classified as a response and smaller reductions or increases considered non‐response. Participants in the standard condition (IG1) received feedback after every session, while those in the guidance‐on‐demand condition (IG2) could reach out to e‐coaches as needed. The basic condition (IG3) involved no additional coaching.

Abbreviation: iCBT‐I, internet‐based cognitive behavioural therapy for insomnia.

### 3.1 Research Question 1: Do Participants Consistently Adhere to SRT?

#### 3.1.1 Rejected SRT From the Beginning (*n* = 2)

Two participants (IG1 = 1 and IG3 = 1) rejected SRT from the beginning. One participant expressed reluctance due to previous negative experiences with SRT, while the other participant expressed concerns that they were already experiencing insufficient sleep and anticipated that SRT might exacerbate their existing stress levels.

So, when it came to reducing the sleep time, that is, radically shortening the time spent in bed, I couldn't do that because I had already been sleeping only three to four hours per night. And I felt that it would create even more pressure if I were to say, okay, I will really get up at night and do something else because I was already completely exhausted and couldn't do it at that moment.

IG3_F_20_rejected.

#### 3.1.2 Discontinued Adhering to SRT (*n* = 8)

Eight participants (IG1 = 3, IG2 = 3 and IG3 = 2) discontinued the implementation of SRT prematurely. They named various reasons for the discontinuation of SRT, including negative effects such as extreme fatigue/impact on daytime functioning, lacking compatibility with daily and occupational life, the impression that SRT was counterproductive, the onset of a COVID infection, and fear of relapsing into a depressive episode.

Well, if I were on vacation, then I could definitely give it a try. But in the professional world, I found that particularly challenging, and I have tried it for about a week and then stopped.

IG1_M_30_discontinued.

#### 3.1.3 Inconsistent Adhering to SRT (*n* = 2)

Two participants (both IG1) described their implementation of SRT, suggesting they understood the concept of SRT and attempted to adhere to it generally but made significant modifications to the prescribed sleep window.

I didn't—and couldn't—accept the procedure, to be honest, because I was so exhausted at times that I simply, I would say, ignored the instructions. But I also tried to have a shorter sleep window so that I could sleep better. There is this theory as it is described. The shorter you sleep, the deeper and better you sleep. That's what I did to some extent…

IG1_F_60_inconsistent.

#### 3.1.4 Consistent Adhering to SRT (*n* = 11)

Eleven participants (IG1 = 7, IG2 = 3, IG3 = 1) described their implementation of SRT in a manner suggesting they adhered to the prescribed sleep window, with only slight modifications.

That's when the sleep restriction started. And that was and is still a huge challenge for me. I started with a lot of discipline. I stuck to the suggested times.

IG2_F_50_consistent_1.

Table 2 summarises the identified hindering and facilitating factors for the adherence to SRT in iCBT‐I, addressing research questions 2 through 5.

**TABLE 2:** Summary of hindering and facilitating factors to the adherence of SRT.

(Sub‐) category	Hindering factors	Facilitating factors
Individual level		
Motivation, commitment personal characteristics	Docility Low frustration tolerance	High level of commitment
Burden of insomnia		High burden of insomnia
Comorbid conditions	Symptoms hindering the implementation of SRT (Perceived) deterioration of comorbid conditions due to SRT	
Occupation	Perceived incompatibility with SRT	Flexibility to adjust occupational activities
Attitudes towards and previous experiences with treatment components	Previous negative experiences with SRT	Previous positive experience with SRT
Attitudes towards the treatment setting	Negative attitude towards iCBT‐I	
Social system	Social burdens Scepticism from social surroundings towards iCBT‐I Lack of social support	Social support
Intervention level		
Individual fit	Perceived lack of individual fit	Perceived individual fit
Information about SRT	Feeling insufficiently informed about SRT	
Sleep diary	Feedback leads to frustration Filling out the sleep diary is too strenuous	Feedback highlights improvements
Session frequency	Perceived as too fast	Fits with individual needs
Guidance from e‐coach		
Support	Needing more support than provided	Feeling well supported Knowing that one can ask e‐coach for support
Commitment		If e‐coach increases commitment
Motivation		If e‐coach increases motivation
Effects		
Negative effects	Pronounced anticipation of negative effects	
Positive effects	Absence of positive effects	Positive effects occurring soon after the introduction of SRT Experiencing small improvements

Abbreviations: iCBT‐I, internet‐delivered cognitive behavioural therapy for insomnia; SRT, sleep restriction therapy.

### 3.2 Research Question 2: Which Individual Factors Hinder and Foster Adherence to SRT?

#### 3.2.1 Motivation, Docility, and Frustration Tolerance

General statements regarding motivation did not show any specific patterns regarding adherence to SRT. However, five participants (IG1 = 3 and IG2 = 2) made statements that were indicative of high levels of commitment, and all of these participants adhered to the SRT consistently.

What steps are still ahead for me, and what can I take away from this? And yes, I'm the kind of person who, once I start something, will see it through to the end.

IG1_F_30_consistent

Four participants (IG2 = 2 and IG3 = 2) made statements indicating high levels of docility (i.e., tendency to align one's behaviour with perceived norms without resistance), and all of them discontinued the SRT.

I really tried to do everything properly. I am always a good patient. I try to do everything until I realize I can't go on anymore.

IG2_F_50_discontinued

Four participants (IG1 = 3, IG2 = 1), who either rejected the SRT (*n* = 1) or discontinued it (*n* = 3), stated that they have low levels of frustration tolerance and patience.

I have a relatively low frustration tolerance. When I think: Oh no. I already know that. Or it doesn't work for me anyway.

IG1_F_40_rejected

Furthermore, statements that indicated discipline and curiosity did not show any patterns regarding adherence to SRT.

#### 3.2.2 Burden of Insomnia

Statements reflecting burden from the insomnia disorder were categorised into moderate, high, and very high levels of distress. Seven participants (IG1 = 3, IG2 = 2 and IG3 = 2) were categorised as experiencing moderate distress (discontinued = 4, inconsistent = 1, consistent = 2). Seven participants (IG1 = 3, IG2 = 3 and IG3 = 1) indicated high distress (rejected = 2, discontinued = 2 and consistent = 3) and six participants (IG1 = 4 and IG2 = 2) indicated very high distress (discontinued = 1 and consistent = 5).

But also because, for me, it was like the last little thread I was clinging to. Because I was really desperate, not knowing what else I could do. Being unable to sleep in your mid‐20s is somehow a challenging issue. That's why I approached it very intensively and really tried to integrate it, to give the whole thing a chance, to be open.

IG1_F_20_consistent_2 (very high burden)

#### 3.2.3 Comorbidities

Five participants (IG1 = 2, IG2 = 2 and IG3 = 1) reported that they currently have or have had depressive symptomatology (discontinued = 3 and consistent = 2). One person who discontinued the SRT stated that the negative effects experienced during the SRT felt like a relapse to a depressive episode.

I was completely worn out, mentally, not even just physically exhausted. But then I said, I‐, because I know what it's like when you drift into such a depressive phase. I didn't want to continue with that. So, I decided to discontinue.

IG3_F_50_discontinued

Another participant who discontinued the SRT noted that the current depressive symptomatology was a barrier to the implementation of the iCBT‐i in general.

I felt so bad at that time that I was relieved if I managed to do my personal care and everything in the morning. And just somehow managed to get my meals together. If I had set additional goals for myself, it would have increased the pressure even more.

IG2_F_50_discontinued

Two participants (IG1 = 1 and IG2 = 1) indicated that they were affected by a trauma‐associated disorder; one of the participants discontinued the SRT, and one rejected the SRT. Four participants (IG1 = 3, IG2 = 1; discontinued = 1, inconsistent = 2 and consistent = 1) indicated that they were suffering from comorbid sleep disorders (i.e., restless legs syndrome and sleep apnea). Two participants (IG1 = 1 and IG2 = 1) who both discontinued the SRT reported that they were suffering from pain symptoms. One of these participants mentioned that the SRT aggravated these symptoms.

#### 3.2.4 Compatibility With Occupational Life

Five participants (IG1 = 3 and IG3 = 2), who did not consistently adhere to SRT, made statements indicating that they did not find the SRT compatible with their occupational life (rejected = 1, discontinued = 3 and inconsistent = 1). Four participants (IG1 = 3 and IG3 = 1) stated that the SRT influenced their occupational activities but that they still implemented the SRT (all consistent). Four participants (IG1 = 3 and IG2 = 1) made statements suggesting that they found it helpful that they were flexible in adjusting their occupational activities during the SRT (discontinued = 1 and consistent = 3).

#### 3.2.5 Previous Experiences With SRT

Three participants (IG1 = 2 and IG3 = 1) had prior experiences with SRT. Two of them saw this as confirmation to undergo it (inconsistent = 1 and consistent = 1), while the other one refused to try out SRT again (rejected = 1).

#### 3.2.6 Attitudes Towards the Treatment Setting

Four participants (IG1 = 3, IG1 = 1; inconsistent = 1, consistent = 3) made statements suggesting they positively evaluated the digital setting, whereas two participants (IG1 = 2; discontinued = 1, inconsistent = 1) indicated a neutral to positive attitude towards the digital setting. On the contrary, four participants (IG1 = 2, IG3 = 2; rejected = 2, discontinued = 2) indicated that they would have preferred a face‐to‐face treatment. In fact, both participants who rejected the SRT (IG1 = 1, IG3 = 1) expected that the treatment would have been delivered in another setting.

Yes, I had thought that it was getting closer to a therapy. Or a support group.

IG1_F_40_rejected

#### 3.2.7 Social System

Three participants (IG1 = 2, IG3 = 1; discontinued = 2 and inconsistent = 1) reported experiencing significant social burdens during the intervention period, including caregiving responsibilities, a relative's cancer diagnosis, and relationship conflicts. Additionally, three participants (IG1 = 1, IG2 = 1 and IG3 = 1; all discontinued) described encountering scepticism from their social system regarding iCBT‐I and the lack of social support.

And during the whole thing, sometimes, well, my partner would say, ‘Why are you sitting at the computer again?’.

IG1_F_60_discontinued

In contrast, two participants (IG1 = 1 and IG2 = 1; both consistent) noted positive support from their social system for the general participation in iCBT‐I, and three participants (IG1 = 2 and IG2 = 1; all consistent) noted support from their social system for the implementation of the SRT.

…when it was really bad, when I just couldn't go on anymore. And then they said, ‘You've come so far already; now you can make it through the two weeks. You can finish it.’ Yeah, so I received a lot of support from my surroundings at that point, and I knew that they would help me if I couldn't go on anymore. And, of course, it gives you that extra push to keep going.

IG2_F_50_consistent _1

Three participants (IG1 = 3; inconsistent = 1, consistent = 2) mentioned that it was difficult to implement their sleep window because of their partner.

So, especially during the sleep time reduction, that was intense because of my wife. (laughter) She was always in bed earlier. And she didn't like that I went to bed so late.

IG1_M_60_consistent

### 3.3 Research Question 3: Which Intervention‐Related Factors Hinder and Foster Adherence to SRT?

#### 3.3.1 Individual Fit of the iCBT‐I

Six participants who did not consistently adhere to SRT (IG1 = 2, IG2 = 2 and IG3 = 2; rejected = 1, discontinued = 4 and inconsistent = 1) expressed that they felt the iCBT‐I did not align with their individual needs.

So, somehow, I couldn't implement [the iCBT‐I] well. [The iCBT‐I] was somehow too theoretical for me, and it didn't really fit into my life. It was a bit, well, not made for me.

IG3_F_50_discontinued

In contrast, four participants (IG1 = 2 and IG2 = 2) who consistently adhered to the SRT indicated that the iCBT‐I aligned with their individual needs.

I really liked the structure, and the content appealed to me. For me, it was perfectly tailored to fit my needs.

IG1_F_60_consistent

#### 3.3.2 Information About SRT

Regarding the SRT, four participants who discontinued SRT (IG1 = 1, IG2 = 2 and IG3 = 1) mentioned that the program provided insufficient information on when the SRT would end again. Moreover, they would have appreciated more support on how to occupy their time during SRT and how to implement SRT during daylight savings.

That really bothered me. I didn't know when it would stop; the recommendation was to continue with the sleep restriction. It was just extended for another week and then another week. I had the feeling at one point that it was like there was a carrot being dangled in front of me, like with a donkey.

IG2_F_50_discontinued

#### 3.3.3 Sleep Diary

Five participants (IG1 = 3, IG2 = 1 and IG3 = 1; consistent = 2, inconsistent = 1, discontinued = 2) expressed that they found the feedback provided through the sleep diary helpful, whereas three participants (IG1 = 1, IG2 = 1 and IG3 = 1; rejected = 2, discontinued = 1) found the feedback to be frustrating. Eight participants (IG1 = 4, IG2 = 2 and IG3 = 2; rejected = 1, discontinued = 4, inconsistent = 2, consistent = 1) mentioned that filling in the sleep diary was too strenuous (e.g., they did not want to do it every day, or they wished for simplifications or technical support). Four participants (IG1 = 1, IG2 = 2 and IG3 = 1; discontinued = 1, consistent = 3) appreciated the option to fill out the sleep diary via a smartphone application instead of the browser‐based version.

#### 3.3.4 Suggested Session Frequency

Five participants (IG1 = 3 and IG2 = 2; all consistent) reported completing the iCBT‐I following the suggested session frequency, thus one session per week. Six participants (IG1 = 4 and IG2 = 2; discontinued = 1, inconsistent = 1, consistent = 4) made statements implying that they valued the possibility of completing the iCBT‐I at their own pace or being able to interrupt it in the case of holidays or other circumstances. Four participants (IG1 = 2, IG2 = 1 and IG3 = 1; discontinued = 2, inconsistent = 1, consistent = 1) stated that the suggested session frequency was too fast to implement the treatment contents intensively. In this context, one participant (IG1, consistent) mentioned that they took over 4 months to complete the two sessions on SRT.

I simply wanted to follow and complete the treatment at my own pace… I wanted to incorporate [the SRT] correctly into my life, make it a habit. That's why I took four months… The reason for that was precisely because I wanted to integrate it properly into my life rhythm.… And I believe I have succeeded because, as I told you, I can now wake up earlier… If I had started the next session right away, maybe I wouldn't have immediately grasped it, how can I say? The principles of this sleep restriction, incorporated them, and continued with the treatment without understanding what the principles were.

IG1_M_40_consistent

### 3.4 Research Question 4: How Does Guidance Through an E‐Coach Influence Adherence to SRT?

#### 3.4.1 Support

Nine participants (IG1 = 6, IG2 = 2 and IG3 = 1) commented that they felt well supported by their E‐coach (discontinued = 1, inconsistent = 1 and consistent = 7). The statements of six participants (IG1 = 3, IG2 = 3; discontinued = 3, inconsistent = 1 and consistent = 2) indicated that they did not see any particular relevance in the e‐coaching. Five participants (IG1 = 1, IG2 = 2 and IG3 = 2; discontinued = 3, consistent = 2) stated that they would have liked more intensive guidance from the e‐coach during SRT (often in the modality of an additional video call). Two individuals who discontinued SRT and were in IG3 reported feeling left alone during the implementation of SRT. They perceived the program's tips as too trivial and expressed needing more guidance, especially when things were not going well.

What the program failed to achieve… it completely left me alone with [the SRT]. I couldn't handle it on my own. I could have only done that in a dialogue with a person or something; with the app, it was just too trivial. Yes. Just go to bed later and then with trivial tips on how to do it.

IG3_F_60_discontinued

The statements of two individuals in IG2 indicated that they were unaware they could ask their e‐coach for additional support (discontinued = 1 and consistent = 1). Two other participants of IG2 (both discontinued) stated that they would have needed more support but did not ask for it, even though they were aware of the option of asking for support.

That I didn't make full use of the coach is actually my own thing. It might have been a bit silly. I could have really benefited more from it.

IG2_M_50_discontinued

In contrast, three participants of the IG2 indicated that they sometimes asked their e‐coach for support (discontinued = 1, consistent = 2). Thirteen participants (IG1 = 10 and IG2 = 3) explicitly stated they knew they could contact the e‐coach (discontinued = 4, inconsistent = 1 and consistent = 8).

…with the knowledge in the back of my mind that there's always someone available as a contact person where I can ask questions. I think I somehow used that once or twice, exactly.

IG2_F_20_consistent

#### 3.4.2 Motivation and Commitment

Twelve participants (discontinued = 3, inconsistent = 1 and consistent = 8), of whom most were in IG1 (IG1 = 8, IG2 = 2 and IG3 = 2), stated that the e‐coach played a role in their motivation. Four participants (IG1 = 3, IG2 = 1; rejected = 1 and consistent = 3) made statements indicating that the commitment to adhere to the intervention was fostered by having an e‐coach. One participant, who was in IG3 and discontinued the implementation of the SRT, indicated that the lack of having somebody to talk to resulted in lower levels of commitment.

I know I have to deliver. So, that has been an important aspect for me. It prevented me from deviating too quickly because I knew there would be a kind of conversation, but in a good way.

IG1_F_60_consistent

### 3.5 Research Question 5: What are the Consequences of the Implementation of SRT (e.g., Negative Effects, Positive Effects, and Influence on Attrition)?

#### 3.5.1 Expectations/Information About Negative Effects

Four participants (IG1 = 3, IG2 = 1; discontinued = 1 and consistent = 3) indicated that the program informed them of potential negative effects during the implementation of SRT. Of these, two participants stated that they felt prepared for possible negative effects through the provided information.

I was able to deal with it quite well because I also thought: Well, these effects are described, even in the program… But I was well prepared for it and could also prepare my family for it.

IG1_M_50_consistent

The two participants (IG1 = 1 and IG3 = 1) who rejected the implementation indicated that they had various expectations regarding negative effects that may occur through the SRT (e.g., higher levels of distress, reduced performance at work, and impairments in roadworthiness).

And I had the feeling that it would build up even more pressure if I were to say to myself now, okay, I'll really get up at night and do something else because, well, I was already completely exhausted and couldn't do that at that time.

IG3_F_20_rejected

One participant (IG3 = 1) who discontinued SRT made a statement indicating that they expected severe negative effects.

I would have had to walk around until a point where I almost fell asleep at the wheel, but I never reached that point.

IG3_F_60_discontinued

Three participants who consistently adhered to the SRT (IG1 = 2 and IG2 = 1) provided statements regarding their expectations of negative effects that may occur during SRT. One participant expressed that they knew it would not be easy, while the other two stated that they expected the negative effects to be more pronounced.

I would have thought. I had thought they would have been worse. But based on my experience, I would say not as bad as I thought.

IG1_M_40_consistent

#### 3.5.2 Experienced Negative Effects

The participants reported the following negative effects: exhaustion, difficulties concentrating, decreased performance, sleepiness, negative impact on the social environment (social withdrawal/increase in conflicts), irritability, negative mood, physical complaints, increased distress, lethargy, dizziness, and migraine. See Supporting Information 5 for an overview of the negative effects experienced and the category‐based evaluation. One participant (IG2, consistent) indicated they experienced almost no impairments through the SRT. Whereas seven participants were categorised as experiencing mild levels of impairment (IG1 = 4 and IG2 = 3; discontinued = 2, consistent =5), and eight participants were categorised as experiencing severe levels of impairment (IG1 = 5, IG2 = 1 and IG3 = 2; discontinued = 2, inconsistent = 1 and consistent = 5).

But I can't even remember that; I don't think I even remember the SRT, which I thought it might get a little bit more difficult. Not even then… That was perfectly fine.

IG2_F_50_consistent_2 (No Impairments)

It was difficult to get through the day. So it was very difficult, yes.

IG1_F_30_consistent (Severe levels of impairment)

#### 3.5.3 Coping Strategies for Negative Effects

The participants mentioned various coping strategies for the negative effects ranging from enduring to social support and drinking caffeine that are listed with the respective counts in Supporting Information 6.

#### 3.5.4 Positive Effects

Six participants (IG1 = 4, IG2 = 1, IG3 = 1; discontinued = 2, inconsistent = 1 and consistent = 3) indicated that they experienced slight improvements in their insomnia symptoms through SRT, whereas nine participants (IG1 = 5, IG2 = 4; discontinued = 3, inconsistent = 1 and consistent = 5) stated that they experienced strong improvements through SRT, which encompassed both sleep‐related benefits (e.g., improved sleep quality, less fragmented sleep) and enhancements in daytime functioning (e.g., better mood, increased energy during the day, being less irritable).

So for me, for example, this sleep restriction therapy worked very well, and I actually noticed improvements during the program. And that's exactly why I was very satisfied with the experience.

IG2_F_20_consistent (Strong improvements)

Three participants (IG1 = 1, IG2 = 1, IG3 = 1; discontinued = 1, consistent = 2) mentioned positive side effects of implementing SRT (e.g., having more time, getting more stuff in the morning done). Two participants (IG1 = 1, IG2 = 1; both consistent) mentioned that small improvements (e.g., the extension of the TIB minutes) motivated them to continue. Three participants (all IG1; all consistent) indicated that the improvements occurred soon after implementing the SRT.

And I really appreciated it because I thought it was really good. And also because I quickly noticed improvements.

IG1_F_20_consistent

#### 3.5.5 Absence of Positive Effects

One person (IG2; discontinued) mentioned that the absence of positive effects of the SRT and the simultaneous monitoring of the sleep diary had a negative impact on their motivation.

So if I'd had values where I thought, ‘Wow, this is going uphill’, then I probably would have felt differently… Only to realize how bad it actually is (laughs). That nothing is happening and that I'm still in a stupid area…

IG2_F_50_discontinued

#### 3.5.6 Influence on Attrition

One participant (IG1, rejected) reported that they discontinued the entire iCBT‐I because they perceived that they could not continue the program once SRT was introduced. Moreover, two participants (IG2 = 1, IG3 = 1; discontinued = 2) stated that they thought about discontinuing the entire iCBT‐I because of difficulties with the SRT.

And when I was really almost ready to stop was relatively early on, when this sleep restriction, this sleep deprivation, had to be carried out.

IG3_F_50_discontinued

## 4 Discussion

This qualitative study investigated barriers and facilitating factors to adherence to SRT in CBT‐I. The interview data suggest that almost half of the participants consistently implemented SRT. However, 9% rejected the implementation of SRT from the beginning, 35% prematurely discontinued SRT, and 9% implemented SRT inconsistently, potentially limiting their ability to benefit fully from this treatment component. Importantly, this categorization was solely based on participants' statements in the interviews and did not incorporate data from sleep diaries. Docility, low levels of frustration tolerance, and previous negative experiences with SRT emerged as hindering individual factors of adherence. In contrast, a high level of insomnia‐related burden, social support, perceived individual fit with the program, positive effects of the SRT, and satisfaction with the therapeutic guidance appeared to foster adherence.

**FIGURE 1:**
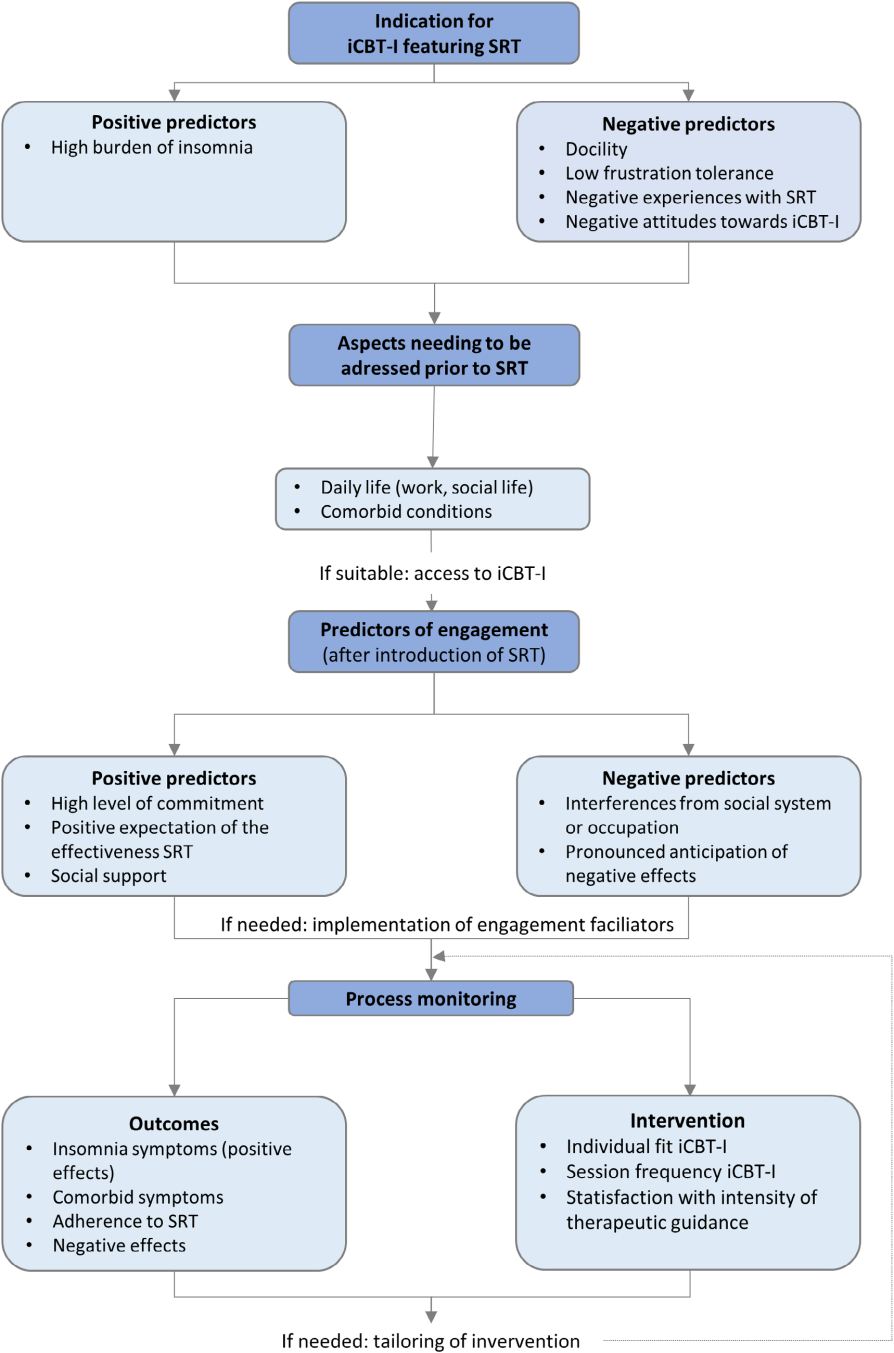
Treatment path model suggesting candidate variables for treatment decisions. The treatment pathway model offers a theoretical framework for the treatment path developed from interview data. The proposed variables serve as candidate variables that, if confirmed in future studies, could inform and guide treatment decisions. iCBT‐I, internet‐delivered cognitive behavioural therapy for insomnia; SRT, sleep restriction therapy.

Based on the study results, we developed a treatment path model for iCBT‐I featuring SRT with candidate hindering and facilitating variables that, if confirmed, could inform and guide treatment decisions. In particular, the model postulates candidate predictors for the indication for SRT in the context of iCBT‐I (i.e., *“Is an iCBT‐I featuring SRT a suitable treatment option for this patient?”*), candidate predictors for treatment engagement (i.e., *“Should engagement facilitators be implemented for this patient?”*), and candidate variables for process monitoring (i.e., *“Does the intervention need to be tailored to the individual needs of the patient?”*). The model is depicted in Figure 1 and described in detail below.

Individual factors that facilitate or hinder the implementation of SRT in iCBT‐I may indicate whether individuals are generally suited for iCBT‐I incorporating SRT. While quantitative studies have not consistently linked baseline insomnia severity with adherence (Agnew et al. 2020; Mellor et al. 2022), our findings suggest that a significant burden associated with insomnia may enhance adherence to SRT. This finding is in line with a study on attrition in iCBT‐I that found that individuals with higher pre‐treatment total sleep time had a higher chance of dropping out (Hebert et al. 2010). It is important to highlight that in the context of the qualitative content analysis, the burden of insomnia was assessed based on participants' statements rather than through a standardised self‐report questionnaire. Moreover, the role of sleep medication use, which 52.2% of participants reported using at least occasionally, was not explored despite its potential to interfere with adherence to SRT (Agnew et al. 2020).

Furthermore, the results of this study show that individuals displaying high levels of docility (i.e., the tendency to align one's behaviour with perceived norms without resistance) may be at risk for discontinuing SRT, potentially reflecting a tendency to prioritise short‐term compliance with external demands (e.g., occupational or social life and initially the instructions of the iCBT‐I) over sustained long‐term commitment to therapeutic requirements. Furthermore, a low frustration tolerance, prior negative experiences with SRT, and a clear preference for face‐to‐face therapy may also have a negative impact on adherence to SRT. If the predictors for indication proposed in this model are confirmed, they could be used to determine whether an iCBT‐I program featuring SRT is a suitable treatment option or if patients should be referred to alternative treatment settings, such as conventional face‐to‐face therapy.

In addition to these candidate predictors for indication, the study suggests that it may also be necessary to consider individuals' current life circumstances and comorbid conditions before assigning them to an iCBT‐I program featuring SRT. A perceived incompatibility between SRT and daily or occupational life appears to hinder adherence to SRT and may be a significant treatment barrier overall. If this incompatibility is temporary—such as stress peaks at occupational projects or planned travels—it may be beneficial to delay the initiation of the iCBT‐I until the current life circumstances change.

While comorbidities are common in insomnia patients, and CBT‐I has been shown to be effective in patients with comorbid conditions (Riemann et al. 2023), our study indicates that comorbid symptoms may be another candidate predictor that might be relevant to consider when assigning patients to iCBT‐I. In their systematic review, Mellor et al. (2022) found that depressive symptoms were significantly negatively associated with adherence to CBT‐I in 50% of the studies. Another review on adherence (Agnew et al. 2020) found that lower levels of depression fostered adherence, but the presence of psychiatric comorbidity was not a significant predictor. According to our study, one potential mechanism by which comorbid symptoms might affect adherence is their impact on participants' ability to engage in SRT. Therefore, it may be necessary to manage symptoms of comorbid conditions (e.g., lack of drive in a depressive episode) if they would hinder the implementation of SRT. However, while quantitative baseline survey data suggested a mild symptom burden for comorbid conditions, it is important to note that the information on comorbidities used in this analysis was derived from self‐reports provided during the interviews. To better understand the role of comorbid conditions on adherence to SRT, future research should further investigate clinically diagnosed comorbidities and their severity as potential predictors.

Predictors of treatment engagement might become apparent shortly after patients are introduced to SRT in iCBT‐I. If candidate predictors are confirmed to be clinically meaningful, these predictors could guide the implementation of engagement facilitators (e.g., additional therapeutic guidance, integrating the iCBT‐I in blended‐care concepts, or providing patients with specific components designed to enhance their motivation to engage in SRT). Patients' commitment and expectations regarding the effectiveness of SRT emerged as candidate predictors for this phase. Furthermore, consistent with previous evidence (Agnew et al. 2020; Mellor et al. 2022), this study suggests that the social system is a candidate predictor for engagement. The results of the present study indicate that social systems can be both a hindering and facilitating factor. Within the context of iCBT‐I as a self‐help intervention, social support may be even more pivotal than in conventional face‐to‐face CBT‐I. Given the existing evidence base, exploring how iCBT‐I could utilise social systems to improve SRT adherence and identify strategies for supporting participants whose social systems do not facilitate SRT implementation appears valuable.

Moreover, this study showed that for some participants, the anticipation of negative effects appears to be a hindering factor for implementing SRT. However, alongside the ethical obligation to inform patients about potential negative effects, this study indicated that such information also helped some patients feel prepared. If future studies confirm the relevance of the anticipation of negative effects, incorporating elements of motivational interviewing (Miller and Rollnick 2012) may benefit patients whose focus on immediate negative effects conceals the potential long‐term benefits of adhering to SRT. Considering negative effects during process monitoring also appears important, as most participants reported mild to severe impairment due to these negative effects. While no clear pattern regarding the experience of negative effects and adherence to SRT emerged, more research in this field is needed to understand the influence of negative effects on the treatment trajectory in iCBT‐I and if certain negative effects may demand the adjustment of the treatment protocol. On the other hand, experiencing positive effects from SRT—both sleep‐related improvements (e.g., improved sleep quality, less fragmented sleep) and improvements in daytime functioning (e.g., better mood, increased energy, reduced irritability)—appears to be an important facilitator for the adherence to SRT. This seems to be especially true when participants notice significant improvements early in the implementation process.

Furthermore, the interview data indicated that the rejection of SRT or difficulties in implementing SRT could negatively impact the course of iCBT‐I. If, indeed, data on adherence to SRT can predict the risk for attrition, this data should be collected during a process monitoring to guide decisions on individual tailoring of iCBT‐I. For example, it could be considered if patients should be provided with more intensive therapeutic guidance during SRT, an option to bypass SRT or to switch to a milder form of SRT, such as sleep compression (Rosén et al. 2023) or sleep regularisation (Manber et al. 1996). This flexibility could foster trust in the treatment process and potentially encourage participants to revisit SRT at a later stage, providing them with a second opportunity to engage with this aspect of the intervention.

The results of this study suggest that SRT may negatively impact comorbid conditions, such as pain, and that the negative effects of SRT might be perceived as a relapse into a depressive episode, which can also be regarded as negative effects. While there is insufficient research on the potential negative effects of iCBT‐I on comorbid conditions, our results indicate that the trajectory of symptoms of comorbid conditions appears to be a candidate variable for process monitoring. Furthermore, according to our study, the absence of positive effects from SRT on insomnia symptoms can impact adherence to SRT, while improvements soon after the introduction of SRT appeared to foster adherence. Therefore, the trajectory of insomnia symptoms could be considered another candidate variable for process monitoring.

Regarding the intervention‐related factors, this study points to a relationship between the individual fit of iCBT‐I (i.e., the extent to which individuals perceive that iCBT‐I aligns with their individual needs) and adherence to SRT. If the individual fit of iCBT‐I can be confirmed as a variable that should be monitored, future studies could explore if and how the individualisation of iCBT‐I can enhance adherence. Statements regarding the session frequency of the iCBT‐I revealed a notable divergence in individual preferences. All participants who followed the suggested session frequency consistently implemented SRT. However, the interviews also showed that participants valued the flexibility to progress through iCBT‐I at their own pace and that some participants wanted to fully integrate SRT into their lives before advancing to the next session. Future studies may explore whether a divergence from the suggested session frequency should also be considered for process monitoring.

Similar to evidence of intervention completion in internet‐based programs in general (Musiat et al. 2022), the guidance provided by an e‐coach appears to enhance adherence to SRT for many participants. Notably, some participants expressed a desire for more intensive support from their e‐coach, particularly during the initial phase of SRT. This observation raises the question of whether more intensive guidance (e.g., via a video call) during the initial phase of SRT in iCBT‐I would enhance adherence. Interestingly, within the guidance‐on‐demand group (IG2), some participants did not utilise the option to contact their e‐coach despite stating that they would have needed more guidance. Although this observation is based on a small subset of participants, it could indicate that individuals experiencing strong doubts or significant challenges with implementing SRT may struggle to initiate requests for additional guidance and could benefit from proactive outreach from e‐coaches. Based on the study's results, satisfaction with and need for therapeutic guidance is another candidate variable for process monitoring in iCBT‐I.

It is important to recognise that the treatment path model was developed based on participants' opinions. While these insights suggest a potential relationship between the proposed candidate variables and adherence or outcomes in iCBT‐I, quantitative investigations are necessary to confirm these associations. The responses provided in the interviews do not permit causal inferences, and it is also possible that some participants' explanations were more akin to justifications rather than genuine reasons. A potential approach to investigating the proposed model could involve quantitative studies assessing whether factors related to the indication for iCBT‐I featuring SRT (e.g., insomnia severity and frustration tolerance) and aspects that may need to be addressed before the intervention (e.g., occupational activities, clinically confirmed comorbid conditions) are predictive of adherence, as measured by sleep diary data. Following the introduction of SRT, the predictive value of factors influencing engagement (e.g., commitment level and social support) and process monitoring variables (e.g., satisfaction with therapeutic guidance, emergence of comorbid symptoms) could be assessed. One potential method for such investigations could involve combining ecological momentary assessments with sleep diary data, allowing for the tracking of sleep restriction therapy trajectories and examining their relationship with potential influencing factors (Kubiak and Smyth 2019).

Moreover, when interpreting the results of this study and the proposed treatment path model, it is important to keep the limitations of this qualitative study in mind. The categorisation of adherence to SRT, burden of insomnia, and information on comorbidities were based on text segments extracted from interviews. While there is currently no consensus on how to rate adherence to SRT, quantitative data analysis might have yielded a different categorisation. Despite efforts to ensure reflexivity through diverse expertise within the working group, the team was composed entirely of psychologists, which may have limited the reflexivity. Furthermore, while two independent coders coded the interviews, only the first author analysed the coded text segments, which could have introduced bias into the analysis process. There is potential for selection bias, as only 26 out of 55 individuals participated in the interviews, and the targeted ratios for treatment response, gender, and IGs were not achieved. Moreover, while all participants had been diagnosed with insomnia by their general care physician, 30.4% exhibited subthreshold insomnia severity based on the Insomnia Severity Index (Bastien et al. 2001). This substantial proportion of participants with subclinical insomnia presentations may limit the generalisability of the findings to populations with more severe insomnia symptoms typically seen in clinical settings. Furthermore, the interview guide placed greater emphasis on exploring the negative effects of SRT compared to its positive effects. As a result, the findings may present an imbalanced representation of the full range of benefits associated with SRT. Additionally, the interviews followed a semi‐structured format, so only a subset of participants may have raised certain topics. Consequently, while this qualitative study has a large sample size for qualitative studies, it is important to recognise that some themes were explored within a limited number of participants. In summary, these limitations underscore the importance of validating the findings and the proposed treatment path model through further research.

In conclusion, this study shows that a significant portion of individuals face challenges with adhering to SRT, which can also affect the overall course of iCBT‐I. To address this, we developed a treatment path model that, if validated, can help determine which individuals are best suited for iCBT‐I featuring SRT. Additionally, the proposed candidate predictors for treatment engagement and process monitoring could guide the future tailoring of iCBT‐I treatment protocols to improve treatment outcomes.

## Author Contributions

**Laura Simon:** conceptualization, investigation, writing – original draft, methodology, visualization, writing – review and editing, project administration, formal analysis. **Lisa Steinmetz:** investigation, validation, writing – review and editing. **Nils Berghoff:** investigation, writing – review and editing, validation. **Charlotte Rehm:** investigation, validation, writing – review and editing. **Lena‐Marie Neumann:** investigation, validation, writing – review and editing. **Ann‐Marie Küchler:** conceptualization, investigation, methodology, writing – review and editing, project administration. **Dieter Riemann:** funding acquisition, writing – review and editing, conceptualization. **David Daniel Ebert:** funding acquisition, conceptualization, writing – review and editing, project administration. **Kai Spiegelhalder:** conceptualization, funding acquisition, writing – review and editing, project administration. **Harald Baumeister:** conceptualization, funding acquisition, writing – review and editing, supervision, project administration, methodology, resources.

## Ethics Statement

Ethical approval for participation in the main and qualitative studies was obtained from the Ethics Committee of the Medical Center—University of Freiburg.

## Consent

The participants provided informed consent before participating in the study.

## Conflicts of Interest

Dr. Ebert has served as a consultant to/on the scientific advisory boards of Sanofi, Novartis, Minddistrict, Lantern, Schoen Kliniken, Ideamed, and German health insurance companies (BARMER, Techniker Krankenkasse) and a number of federal chambers for psychotherapy. He is also a shareholder of ‘GET.ON Institut für Online Gesundheitstrainings GmbH für Gesundheitstrainings online GmbH’ (HelloBetter), which aims to implement scientific findings related to digital health interventions into routine care and is the provider of the intervention under investigation in this study. All other authors have nothing to report.

## Supporting information


Data S1.

## Data Availability

The data that support the findings of this study are available on request from the corresponding author. The data are not publicly available due to privacy or ethical restrictions.
