# Supplementary material for: Barriers and Facilitators of Sleep Restriction Therapy in Internet‐Delivered CBT‐I: A Qualitative Content Analysis and the Development of a Treatment Path Model
Source: J Sleep Res. 2025 Feb 24;34(6):e70018. doi: 10.1111/jsr.70018 (PMC12592822; doi:10.1111/jsr.70018)
Supplement: Supplementary file 1 — Data S1. [file JSR-34-e70018-s001.docx]

# Barriers and facilitators of sleep restriction therapy in internet-delivered CBT-I: A qualitative study and the development of a treatment path model

# Online Supplementary Materials

**Authors:** Laura Simon^1^, Lisa Steinmetz^2^, Nils Berghoff^1^, Charlotte Rehm^1^, Lena-Marie Neumann^1^, Ann-Marie Küchler^2^, Dieter Riemann^2^, David Daniel Ebert^3,4^, Kai Spiegelhalder^2^, Harald Baumeister^1^

**Affiliations:**

1: Institute of Psychology and Education, Department of Clinical Psychology and Psychotherapy, Ulm University, Ulm, Germany

2: Department of Psychiatry and Psychotherapy, Faculty of Medicine, Medical Centre - University of Freiburg, University of Freiburg, Freiburg, Germany

3: GET.ON Institut für Online Gesundheitstrainings GmbH (operating under the registered brand ‘HelloBetter’), Hamburg, Germany

4: Department of Sport and Health Sciences, Technical University of Munich, Munich,Germany

[Supplement 1: Standards for Reporting Qualitative Research (SRQR) - Checklist 2](#_Toc177723739)

[Supplement 2: Interview guide 5](#_Toc177723740)

[Supplement 3: Description of the categories 15](#_Toc177723741)

[Supplement 4: Participants 22](#_Toc177723742)

[Supplement 5: Negative effects 23](#_Toc177723743)

[Supplement 6: Coping strategies 24](#_Toc177723744)

## Supplement 1: Standards for Reporting Qualitative Research (SRQR) - Checklist

|  | **Standards for Reporting Qualitative Research (SRQR)*** |  |
| --- | --- | --- |
|  | <http://www.equator-network.org/reporting-guidelines/srqr/> |  |
|  |  | **Page/line no(s).** |
| **Title and abstract** | |  |
|  | **Title** - Concise description of the nature and topic of the study Identifying the study as qualitative or indicating the approach (e.g., ethnography, grounded theory) or data collection methods (e.g., interview, focus group) is recommended | Page 0 |
|  | **Abstract** - Summary of key elements of the study using the abstract format of the intended publication; typically includes background, purpose, methods, results, and conclusions | Page 1 |
|  |  |  |
| **Introduction** | |  |
|  | **Problem formulation** - Description and significance of the problem/phenomenon studied; review of relevant theory and empirical work; problem statement | Page 2 |
|  | **Purpose or research questio**n - Purpose of the study and specific objectives or questions | Page 2 |
|  |  |  |
| **Methods** | |  |
|  | **Qualitative approach and research paradigm** - Qualitative approach (e.g., ethnography, grounded theory, case study, phenomenology, narrative research) and guiding theory if appropriate; identifying the research paradigm (e.g., postpositivist, constructivist/ interpretivist) is also recommended; rationale** | Page 4 (Qualitative content analysis) |
|  | **Researcher characteristics and reflexivity** - Researchers’ characteristics that may influence the research, including personal attributes, qualifications/experience, relationship with participants, assumptions, and/or presuppositions; potential or actual interaction between researchers’ characteristics and the research questions, approach, methods, results, and/or transferability | Page 4 (Qualitative content analysis) |
|  | **Context** - Setting/site and salient contextual factors; rationale** | Page 4  (Data collection) |
|  | **Sampling strategy** - How and why research participants, documents, or events were selected; criteria for deciding when no further sampling was necessary (e.g., sampling saturation); rationale** | Page 3-4 (Sampling strategy) |
|  | **Ethical issues pertaining to human subjects** - Documentation of approval by an appropriate ethics review board and participant consent, or explanation for lack thereof; other confidentiality and data security issues | Page 4 (Sampling strategy) |
|  | **Data collection methods** - Types of data collected; details of data collection procedures including (as appropriate) start and stop dates of data collection and analysis, iterative process, triangulation of sources/methods, and modification of procedures in response to evolving study findings; rationale** | Page 4  (Data collection) |
|  | **Data collection instruments and technologies** - Description of instruments (e.g., interview guides, questionnaires) and devices (e.g., audio recorders) used for data collection; if/how the instrument(s) changed over the course of the study | Page 4 (Interview guide + Data collection) |
|  | **Units of study** - Number and relevant characteristics of participants, documents, or events included in the study; level of participation (could be reported in results) | Page 5 (Results) |
|  | **Data processing** - Methods for processing data prior to and during analysis, including transcription, data entry, data management and security, verification of data integrity, data coding, and anonymization/de-identification of excerpts | Page 4-5  (Data collection + Qualitative content analysis) |
|  | **Data analysis** - Process by which inferences, themes, etc., were identified and developed, including the researchers involved in data analysis; usually references a specific paradigm or approach; rationale** | Page 4-5 (Qualitative content analysis) |
|  | **Techniques to enhance trustworthiness** - Techniques to enhance trustworthiness and credibility of data analysis (e.g., member checking, audit trail, triangulation); rationale** | Page 5 (Qualitative content analysis) |
|  |  |  |
| **Results/findings** | |  |
|  | **Synthesis and interpretation** - Main findings (e.g., interpretations, inferences, and themes); might include development of a theory or model, or integration with prior research or theory | Page 6-12 |
|  | **Links to empirical data** - Evidence (e.g., quotes, field notes, text excerpts, photographs) to substantiate analytic findings | Page 6-12 |
|  |  |  |
| **Discussion** | |  |
|  | **Integration with prior work, implications, transferability, and contribution(s) to the field -** Short summary of main findings; explanation of how findings and conclusions connect to, support, elaborate on, or challenge conclusions of earlier scholarship; discussion of scope of application/generalizability; identification of unique contribution(s) to scholarship in a discipline or field | Page 12-16 |
|  | **Limitations** - Trustworthiness and limitations of findings | Page 15 |
|  |  |  |
| **Other** | |  |
|  | **Conflicts of interest** - Potential sources of influence or perceived influence on study conduct and conclusions; how these were managed | Page 0 |
|  | **Funding** - Sources of funding and other support; role of funders in data collection, interpretation, and reporting | Page 0 |
|  |  |  |
|  | **Reference:** |  |
|  | O'Brien BC, Harris IB, Beckman TJ, Reed DA, Cook DA. **Standards for reporting qualitative research: a synthesis of recommendations.** *Academic Medicine*, Vol. 89, No. 9 / Sept 2014  DOI: 10.1097/ACM.0000000000000388 |  |

## Supplement 2: Interview guide

The interview guide was developed to evaluate the barriers and facilitators of the stepped-care model (i.e., qualitative main study) and to answer the specific research questions of this sub-study. All sections of the interview data were considered for the current study. Segments that were irrelevant to the research questions of this sub-study were not coded.

| **Main theme** | **Key question** | **Memo for possible questions** | **Specific questions** |
| --- | --- | --- | --- |
| **Opening question** | You have received treatment for your insomnia disorder as part of GET Sleep. First of all, I would like to ask you to tell me freely how you experienced this time. |  |  |
| **Perceived effectiveness** | How have your sleep problems changed in the last few months? | *If no change:*  To what would you attribute the persistence of your sleep problems?  *If there are changes, possible questions regarding:*   - Quality of sleep - Quantity of sleep (e.g., number of hours, time to fall asleep) - general changes vs. changes on individual days - Impairments throughout the day, e.g., sleepiness, difficulty concentrating |  |
|  | *If change:*  What do you think accounts for the change/improvement/worsening of your sleep problems? | - Treatment as part of GET Sleep - Psychoeducation (GP) - iCBT-I - individual components of the iCBT-I - Specialist treatment | *If improvement:*  You have just mentioned some treatment components that have helped to improve your sleep problems.  Which of these components did you find most helpful?  *If deterioration:*  You have just mentioned some treatment components that have contributed to the worsening of your sleep problems.  Which of these components worsened your sleep problems the most? |
|  |  | Cognition | Every person has different beliefs or attitudes towards sleep.  To what extent has your attitude towards sleep/your sleep problems changed in the last few months? |
|  |  | Behavior | What are your sleeping habits currently?  How have your sleeping habits changed? |
| **Perception treatment process** | We are interested in learning how participants perceived the entire treatment process within GET Sleep.  Please briefly describe your treatment, i.e., what different treatment steps you went through. | Organization | How did you experience the process and organization of the individual treatment steps?  To what extent have you used other forms of treatment? |
|  |  | Contact person | Did you generally feel well looked after if you had any questions or problems?  *If not:*  At what points in the treatment process and for what concerns was this not the case?  *If so:*  What made you feel particularly supported? What contributed to you feeling well looked after? |
|  | How satisfied were you with making appointments, e.g., B. Conversations with your online family doctor or coaching calls with your E-Coach? | Appointments & delays | Did you experience delays in the treatment process? If so, at what point in the treatment process did these occur, and why did they occur? |
|  |  | Miscellaneous | Were there any other difficulties or problems that arose during the course of the treatment? |
|  |  | Evaluation of the stepped-care model | The treatment as part of GET Sleep was structured in such a way that, if desired, you received psychoeducation from your general care physician in the first step, then completed the online training and, if necessary, specialist treatment. How do you rate this stepped-care treatment concept? |
|  |  | Difficulties transitioning | Were there any problems transitioning between the different levels of treatment? |
| **Online platform** | How satisfied were you with the online platform on which the "GET Sleep" training was provided? | - Optics - Find their way - Feel good - Content | How did you find your way around the online platform? (Navigation)  How did you find the visual design of the online platform?  Did you feel comfortable on the online platform?  To what extent were you satisfied with the content of the training? To what extent did you perceive these to be scientifically sound/high quality? |
|  | We would be interested to know how you used the online platform.  To what extent have you implemented the elements that were taught to you as part of GET Sleep in your everyday life? | Usage behavior | How intensively have you been working with the treatment components?  How intensively did you spend *time* dealing with the treatment components? |
| **Therapeutic relationship** | How would you describe your E-Coach? | Features E-Coach | What characteristics did he/she have?  In your opinion, what generally characterizes a good E-Coach? What characteristics should it have? |
|  | How would you read the competence of your E-Coach? | Therapeutic competence E-Coach | Did you have the impression that your E-Coach had a high level of expertise in the area of sleep disorders? |
|  | How did you find the relationship with your E-Coach? How did you feel in contact with your E-Coach? | - Expectations - Sympathy - Appreciation/understanding - Addressing feelings and needs - Impact factors of a good relationship | What expectations did you have regarding contact with your E-Coach?  To what extent were these expectations met or disappointed?  Did you like your E-Coach? Did you feel like he liked you?  To what extent did you feel understood by your E-Coach?  To what extent did you have the impression that your E-Coach was able to respond to your feelings and needs?  *If positive experience:*  What contributed to you being able to build a good relationship with your E-Coach?  What would have helped you build an even better relationship with your E-Coach?  *If negative experience:*  What would have helped you build a better relationship with your E-Coach? / to feel more comfortable with your E-Coach? |
|  |  | Influence of the online setting on the therapeutic relationship | What advantages have you perceived in the therapeutic relationship due to online contact with your E-Coach?  What barriers did you perceive in the therapeutic relationship due to online contact with your E-Coach? |
|  | At what points did you come into contact with your E-Coach? | Individual E-Coaching process |  |
|  | In your opinion, what role does the support of the E-Coach play in the effectiveness of the training and in "sticking with it"? | - Tasks of the E-Coach - Support with therapy goals | What tasks did the E-Coach take on?  To what extent did your E-Coach support you in achieving your therapy goals?  *If good support:*  Which aspects of the support from the E-Coach did you find particularly helpful?  What would you have liked more support from the E-Coach?  *If bad support:*  What would you have liked more support from the E-Coach? |
| **Negative effects** | Negative effects may occur during treatment. We would be interested to know whether you experienced any negative effects during online training. | *Possible areas of impairment:*   - *Somatic/physical complaints* - *Cognitive* - *Sensorimotor (coordination difficulties)* - *Affective/mood* - *Difficulty functioning in everyday life, coping with everyday life (daytime functioning)* - *Difficulties/conflicts in relationships, social situations/activities (social functioning)* - *Productivity at work/performance (work productivity)*   *Responsible therapy component:*   - *Observe sleep* - *Sleep restriction therapy* - *Sleep diary* - *Evening rituals* - *Stimulus control* - *Cognitive restructuring (thought analysis, questioning thoughts, calm thought)* - *Anti-Rumination Strategies* - *Learning relaxation techniques (PMR, autogenic training, mindfulness)* | How impaired did you feel by this?  Which part of the treatment do you associate with the events mentioned? |
|  |  | Reducing bedtime (adjusting the time in bed to the actual amount of sleep) | Did reducing bedtime have a negative impact on you? If so, please describe the effect. |
|  |  |  | For example, did you have difficulty concentrating, did your mood change, did you have physical complaints, or was your performance reduced at work? |
|  |  | Dealing with negative effects | How did you deal with these negative effects? |
| **Questions for dropouts** *(for patients who have completed less than 80% of the online training)* |  | Reasons for discontinuing treatment  Examples   - Impression complaints do not improve - Significant improvement in symptoms - Not enough time - Technical problems - Other treatment | For what reasons did you end the online training early? |
|  |  | Suggestions for improvement/change requests | What would have helped you complete the online training to the end?  What should have been different to complete the online training? |
| Questions for slow processors |  |  | Why did you need more than 12 weeks to complete the online training? |
| Questions for participants completing the intervention in the suggested time frame | What helped you stick with the online training and work through the modules? |  |  |
| **Final question** | We have now reached the end of the questions.  Can you think of anything else that you would like to add to your experience at GET Sleep?  Is there anything else that you wish had been different that hasn't been discussed yet? |  |  |

## Supplement 3: Description of the categories

| Name | Description | Application | Example |
| --- | --- | --- | --- |
| **Adherence to SRT** | | | |
| Rejected SRT from the beginning | Statements indicating that participants refused to implement SRT from the beginning of the iCBT-I. | After reading the entire interview, this category was assigned to participants whose statements conveyed that they rejected the implementation of SRT from the beginning of the iCBT-I. | *“I don't do sleep reduction. Period. It's not an option for me.”*  IG1_F_40_rejected |
| Consistent adhered to SRT | Statements indicating that participants consistently adhered to the SRT as the iCBT-I indicated. | After reading the entire interview, this category was assigned to participants whose statements conveyed that they consistently adhered to the SRT as the iCBT-I indicated. Mild deviations (e.g., taking planned short naps) were allowed. | *“I made sure that I managed to shorten my bedtime. I even paid very, very close attention to it during the program. I actually got up at six at the weekend and did something else.”*  IG2_F_50_consistent_2 |
| Inconsistent adhered to the SRT | Statements indicating that participants understood the concept of SRT and attempted to implement it on a general level, yet making considerable adjustments to the recommended sleep window or statements indicating that participants implemented the SRT only occasionally. | After reading the entire interview, this category was assigned to participants whose statements conveyed that they inconsistently adhered to the SRT as the iCBT-I indicated. Hence, participants made moderate to large alterations (skipping several days of the SRT, adjusting the sleep window by several hours) to the recommended sleep window. | *“I didn't - and couldn't - accept the procedure, to be honest, because I was so exhausted at times that I simply, I would say, ignored the instructions. But I also tried to have a shorter sleep window so that I could sleep better. There is this theory as it is described. The shorter you sleep, the deeper and better you sleep. That's what I did to some extent…”*  IG1_F_60_inconsistent |
| Discontinued SRT | Statements indicating that participants first consistently adhered to the SRT as the iCBT-I indicated it but then discontinued after a short period. | After reading the entire interview, this category was assigned to participants whose statements conveyed that they first implemented the SRT but that they discontinued adhering to the SRT despite indications of the iCBT-I to continue. | *“Well, I tried it out for a week. And I [quit] - because the system then suggested to me that I had to get up at four in the morning.”*  IG3_F_50_discontinued |
| **Individual factors** | | | |
| Motivation, commitment, personal characteristics | Statements indicating the personal states and traits that may influence the completion of the program (e.g., level of motivation, determination to complete the program, docility). | The entire interview was coded for statements conveying the participant's attitude toward completing the iCBT-I. Subsequently, inductively derived subcategories were built (e.g., frustration tolerance and determination). The motivation subcategory was further divided into high and low motivation. | *"I'm ambitious, and I wanted to see it through."*  IG1_F_20_consistent_1 |
| Burden of insomnia | Statements describing the burden resulting from the insomnia disorder (e.g., the urgency of requiring treatment and the impact of the disorder on daily functioning). | The entire interview was coded for statements conveying the burden of insomnia. Subsequently, these codes were systematically categorized for each participant into moderate, high, or very high burden of insomnia. | *“So for me personally, that was kind of the very last step where I knew, okay, I can do something.”* IG1_F_20_consistent_2 |
| Comorbidities | Statements indicating that patients were suffering from other mental or somatic illnesses and how these comorbidities affect the adherence to the SRT. | All coded segments of the category comorbidity were reviewed to build clusters (e.g., pain symptoms, depression). | *“I was completely worn out, mentally, not even just physically exhausted. But then I said, I-, because I know what it's like when you drift into such a depressive phase. I didn't want to continue with that. So, I decided to discontinue.”*  IG3_F_50_discontinued |
| Occupation | Statements pertaining to the compatibility of occupational life and the adherence to SRT | The entire interview was coded to capture statements regarding the compatibility of SRT with participants' occupational life. Following this, three inductively subcategories were derived from these codes. Participants were then assigned to one of the following codes: SRT incompatible with professional activities, SRT affects professional activity but remains feasible, or professional activities were adjusted during SRT. | *“But it's not feasible in my everyday life as a teacher. And as a commuter...I'll just have an accident if I go down any further [with my sleep window]. Then I'd have to take sick leave.”*  IG1_F_40_rejected |
| Attitudes towards and previous experiences with treatment components | Statements indicating that participants had earlier experiences with SRT and how these experiences were affecting the implementation of SRT, as well as statements regarding attitudes towards treatment components other than SRT. | The entire interview was coded to capture statements regarding attitudes towards and previous experiences with treatment components. | *“Because I'm not the type for that, exactly. And that's why I would say just exercises, muscle relaxation, I think progressive muscle relaxation was one chapter. The other was these mindfulness exercises. Exactly. Or mediation, that's just not my thing, which I would do regularly.”* IG1_F_20_consistent_2  *“This sleep restriction really got on my nerves back then in rehab.”* IG3_F_50_consistent |
| Attitudes towards the treatment setting | Statements regarding the attitudes to the treatment setting or preference for other treatment settings (e.g., face-to-face therapy). | The entire interview was coded to capture statements regarding the attitude toward the treatment setting. Following this, inductive subcategories (i.e., positive evaluation of online setting, neutral to positive evaluation of online setting, preferences for face-to-face therapy). | Positive to neutral: *“It has to be said that online forms like this have big advantages but also a few small disadvantages. The big advantage is that you can do it anytime and anywhere. And you don't always have to be tied to one person to get content in the first place. The disadvantage is that you're not forced to take your time but can always do it as a stopgap whenever it suits you. And that, on the other hand, you also have a bit of an off-the-peg treatment, to put it colloquially.”* IG1_M_30_inconsistent |
| Social system | Statements concerning the social system, including its supportive or stress-inducing nature, and how it influenced the implementation of SRT. | The entire interview was coded to capture statements regarding the social system of the participants. Subsequently, inductively subcategories were derived to capture social burdens, support or lack of support in the implementation of the iCBT-I and SRT, and the influences of the sleep window on the partner. | Social support in the implementation of SRT: *“I had a lot of support from those around me. I think that's what got me through. So yes, without my work colleagues, friends, neighbors, and so on, I wouldn't have been able to do it, yes.”* IG2_F_50_consistent _1 |
| **Intervention factors** | | | |
| Individual fit iCBT-I | Statements pertaining to information to which extent participants perceived that the iCBT-I aligned with their individual needs and life circumstances. | The entire interview was coded to capture statements regarding the individual fit of the iCBT-I. Based on these coded segments, the participants were then assigned whether the iCBT-I did align or did not align with their individual needs. | iCBT-I did not align: “*So, somehow, I couldn't implement [the iCBT-I] well. [The iCBT-I] was somehow too theoretical for me, and it didn't really fit into my life. It was a bit, well, not made for me.”* IG3_F_50_discontinued  iCBT-I did align: *“I really liked the structure, and the content appealed to me. For me, it was perfectly tailored to fit my needs.”* IG1_F_60_consistent |
| Information about SRT | Statements about the information on the implementation of the SRT were provided by the iCBT-I, and the questions remained open. | The coded segments were reviewed to evaluate whether participants required additional information on the implementation of SRT and, if so, to identify the specific details they would have needed. | *“That really bothered me. I didn't know when it would stop, the recommendation to continue with the sleep restriction. It was just extended for another week and then another week. I had the feeling at one point that it was like a carrot being dangled in front of me, like with a donkey.”* IG2_F_50_discontinued |
| Sleep diary | Statement concerning the sleep diary (e.g., if it was helpful/ strenuous, what modality was provided). | The coded segments were examined to assess participants' experiences with the sleep diary and to identify suggestions to enhance the sleep diary's usability. | *“The data helped me to gain insight. So there's no question about it. And I would say that logging your sleep is an important part of it. But it has to be so simple that it's not such an evil, so if you can quickly say in the morning, what do I know, "Siri, Hello Better. Seven o'clock." Or something like that instead of struggling through this app, maybe the chances are better. At some point, I no longer had the energy or motivation to log my life.”* IG3_F_60_discontinued |
| Session frequency | Statements on the session frequency of the iCBT-I (e.g., to what extent the session frequency was perceived as appropriate) and also the time frame in which the modules were completed | The coded segments were reviewed to classify participants into three groups: those who adhered to the recommended pace while completing the iCBT-I, those who expressed satisfaction with the option to progress at their own pace, and those who indicated that the session frequency was too rapid for their preference. | *“Well, if I remember correctly, I think the units were once a week at the beginning. And I thought that was too often. I felt that was too often. I would have preferred once every ten days or even once every two weeks so that you could really try out the new methods in different everyday situations. Sometimes, you're doing well; sometimes, you're not doing so well. And then, depending on whether the method works better or worse, or whether you have additional questions. So I didn't think that was so good; for me personally, it was just too quick.”* IG1_M_30_discontinued |
| **E-Coaching** | | | |
| Support | Statements pertaining to information on how participants experienced the support (e.g., quality and intensity) that they received from the E-Coaches. | The entire interview was coded to capture statements regarding how participants experienced the support from the E-Coach.  Inductive subcategories were built to categorize participants who reported feeling well-supported, those who did not perceive relevance in E-Coaching, and those expressing a desire for more intensive E-Coaching during the SRT. For participants of IG2, the circumstances under which participants utilized coaching on demand were explored. For participants of IG3, it was explored whether participants expressed a need for more support. | *What the program failed to achieve... it completely left me alone with [the SRT]. I couldn't handle it on my own. I could have only done that in a dialogue with a person or something; with the app, it was just too trivial. Yes. Just go to bed later and then with trivial tips on how to do it.”*IG3_F_60_discontinued |
| Commitment | Statements pertaining to information on how the E-Coaching influenced the commitment to adhere to the iCBT-I. | The entire interview was coded to capture statements regarding whether participants had the perception that the presence of an E-Coach (e.g., coaching calls and feedback) influenced their commitment. |  |
| Motivation | Statements pertaining to information on how the E-Coaching influenced the participant's motivation. | The entire interview was coded to capture statements regarding whether participants had the perception that the presence of an E-Coach influenced their motivation. These coded segments were used to categorize participants who stated that the E-Coach played a role for their motivation to complete the iCBT-I. | “But I said - I said, she read my stuff and my things straight away. And that's how I got the impression, and so I said, you keep going. She actually motivated me to keep going.”IG1_F_60_discontinued |
| **Effects** | | | |
| Information on negative effects/ expectations of experiencing negative effects | Statements pertaining to information on how well participants felt informed regarding negative effects that may occur through the implementation of SRT, along with their expectations regarding the severity and impact of such negative effects. | The entire interview was coded to capture statements regarding information and expectations of negative effects that may occur as a result of the SRT. Based on these coded segments, it was analyzed whether the information about negative effects prepared participants for the implementation of SRT, whether the expectation of negative effects deterred the implementation of SRT, and whether the anticipated negative effects aligned with the actual experienced negative effects. | *“I was able to deal with it quite well because I also thought: Well, these effects are described, even in the program... But I was well prepared for it and could also prepare my family for it.”* IG1_M_50_consistent |
| Experience of negative effects | Statements pertaining to information on actual experience of negative effects that were attributed to the SRT (e.g., type of negative effects experienced and the resulting impairment). | The entire interview was coded for statements conveying information on the experience of negative effects that were attributed to the SRT. Subsequently, inductively derived subcategories were established to cluster negative effects (e.g., exhaustion, irritability). Using the statements conveying information on the impairments resulting from these negative effects, participants were categorized based on experiencing almost no impairments, mild levels of impairment, or severe levels of impairment. | *“Would say no significant negative effects. Because as I said, the negative effect at the end was just, tiredness and distraction during the day after starting the sleep restriction therapy, shall we say…. Of course, you feel more tired the next day. You feel in a bad mood, that can also be a negative effect. Then work, your, I would say your rhythm, your, doing things, doing the work was slower of course. Because your reactivity wasn't like it used to be, because yes, you're tired, you're sleepy. So. One, but I would say I experienced these negative effects for about a month... Then it settled in.”* IG1_M_40_consistent |
| Coping strategies | Statements addressing how participants coped with the occurrence of negative effects. | The entire interview was coded for statements conveying information on how participants coped with the occurrence of negative effects. Subsequently, inductively derived subcategories were established to cluster coping strategies. | *“For example, one tip was when it was really hard for me when my eyes would close because I was too tired. I would go for a little walk. I would have a coffee or whatever. I followed these instructions from the training sessions. And it helped me, I would say.”* IG1_M_40_consistent |
| Positive effects | Statement on improvements on positive effects (e.g., timing and extent improvement of the insomnia symptoms, positive side effects) that participants attributed to the SRT. | The entire interview was coded for statements conveying information on the positive effects of the SRT. Subsequently, the codes were categorized into participants experiencing slight or strong improvements. Furthermore, statements on positive side effects and the influence of positive effects were analyzed. | *“So for me, for example, this sleep restriction therapy worked very well, and I actually noticed improvements during the program. And that's exactly why I was very satisfied with the experience.”* IG2_F_20_consistent |
| Absence of positive effects | Statement on the absence of positive effects of the SRT. | The entire interview was coded for statements conveying information on the absence of positive effects of the SRT. | *“So if I'd had values where I thought, "Wow, this is going uphill", then I probably would have felt differently…. Only to realize how bad it actually is ((laughs)). That nothing is happening and that I'm still in a stupid area….”* IG2_F_50_discontinued |
| Influence on attrition/ thoughts about attrition | Statement pertaining information of the influence of SRT on attrition (e.g., thoughts about discontinuing the entire iCBT-I, actually discontinuing the iCBT-I). | The entire interview was coded for statements conveying information on how the experience of the SRT influenced thoughts on discontinuing iCBT-i as the whole. | “*And where I was really almost ready to stop was relatively early on, when this sleep reduction, this sleep deprivation, had to be carried out*.” IG3_F_50_discontinued |
| *Comment.* iCBT = internet-delivered cognitive behavioral therapy for insomnia. SRT = sleep restriction therapy.  The main categories adherence to SRT, individual factors, intervention factors, e-coaching, and effects were deductively derived. Subcategories within these main categories were identified inductively from the coded text segments. | | | |

## Supplement 4: Participants

| **Participant** | **IG** | **Gender** | **Age** | **Adherence to SRT** | **Dropout** | **Insomnia**  **Severity T0** | **Insomnia**  **Severity T3** | **Responder** |
| --- | --- | --- | --- | --- | --- | --- | --- | --- |
| IG3_F_50_discontinued | 3 | W | 50-59 | discontinued | completer | moderate | moderate | no |
| IG2_F_50_discontinued | 2 | W | 50-59 | discontinued | completer | subthreshold | subthreshold | no |
| IG2_M_50_discontinued | 2 | M | 50-59 | discontinued | dropout | moderate | moderate | no |
| IG2_F_50_consistent _1 | 2 | W | 50-59 | consistent | completer | moderate | none | yes |
| IG1_M_40_consistent | 1 | M | 40-49 | consistent | dropout | subthreshold | subthreshold | no |
| IG1_F_60_discontinued | 1 | W | 60 | discontinued | completer | subthreshold | moderate | no |
| IG1_F_20_consistent_1 | 1 | W | 20-29 | consistent | completer | subthreshold | subthreshold | no |
| IG3_F_20_rejected | 3 | W | 20-29 | rejected | dropout | moderate | none | yes |
| IG3_F_60_discontinued | 3 | W | 60 | discontinued | dropout | moderate | none | yes |
| IG1_M_30_discontinued | 1 | M | 30-39 | discontinued | dropout | moderate | subthreshold | yes |
| IG1_W_30_discontinued | 1 | W | 30-39 | discontinued | completer | subthreshold | subthreshold | no |
| IG1_M_60_consistent | 1 | M | 60 | consistent | completer | subthreshold | subthreshold | no |
| IG1_F_30_consistent | 1 | W | 30-39 | consistent | completer | moderate | moderate | no |
| IG1_F_60_inconsistent | 1 | W | 60 | inconsistent | completer | moderate | moderate | yes |
| IG1_F_40_rejected | 1 | W | 40-49 | rejected | completer | severe | subthreshold | yes |
| IG1_F_60_consistent | 1 | W | 60 | consistent | completer | moderate | none | yes |
| IG2_F_20_consistent | 2 | W | 20-29 | consistent | completer | moderate | subthreshold | yes |
| IG3_F_50_consistent | 3 | W | 50-59 | consistent | completer | severe | severe | no |
| IG2_F_50_consistent_2 | 2 | W | 50-59 | consistent | completer | severe | moderate | no |
| IG1_F_20_consistent_2 | 1 | W | 20-29 | consistent | completer | moderate | none | yes |
| IG2_F_50_discontinued | 2 | W | 50-59 | discontinued | completer | moderate | subthreshold | no |
| IG1_M_50_consistent | 1 | M | 50-59 | consistent | completer | severe | subthreshold | yes |
| IG1_M_30_inconsistent | 1 | M | 30-39 | inconsistent | dropout | subthreshold | moderate | no |
| *Comment.* IG = Intervention group. Participants in the standard condition (IG 1) received feedback after every module, while those in the guidance-on-demand condition (IG2) could reach out to E-Coaches as needed. The basic condition (IG3) involved no additional coaching. Rejected =participant rejected SRT from the beginning, discontinued = participants discontinued SRT prematurely, inconsistent = participants inconsistently adhered to SRT, consistent participants consistently adhered to SRT. Completers were defined as participants who completed at least 80% of the internet-based cognitive behavioral therapy for insomnia within 12 weeks, while dropouts completed less than 80% within the same timeframe. Insomnia severity was assessed using the Insomnia Severity Index (Bastien et al., 2001) and categorized according to the sum score into none (0–7), subthreshold (8-14), moderate severity (15-21), severe (22-28). Response was determined based on the Insomnia Severity Index score six months after study inclusion, with a reduction of 8 points or more classified as a response and smaller reductions or increases considered non-response. | | | | | | | | |

## Supplement 5: Negative effects

| **Negative Effects** | **Number of participants** | **Intervention groups** | | | **Adherence to SRT** | | |
| --- | --- | --- | --- | --- | --- | --- | --- |
|  |  | **IG1** | **IG2** | **IG3** | **Discontinued** | **Inconsistent** | **Consistent** |
| Exhaustion | 14 | 9 | 5 | 1 | 6 | 1 | 8 |
| Difficulties concentrating | 11 | 7 | 3 | 1 | 3 | 1 | 7 |
| Decreased performance (e.g., at occupation) | 9 | 6 | 2 | 1 | 1 | 0 | 8 |
| Sleepiness | 7 | 2 | 3 | 2 | 3 | 0 | 4 |
| Negative impact on the social environment (social withdrawal or increase in conflicts) | 8 | 4 | 3 | 1 | 2 | 0 | 6 |
| Irritability | 7 | 5 | 1 | 1 | 2 | 0 | 5 |
| Negative mood | 5 | 3 | 1 | 1 | 2 | 0 | 3 |
| Physical complaints | 5 | 2 | 2 | 1 | 1 | 1 | 3 |
| Distress/feeling under pressure | 3 | 1 | 1 | 1 | 2 | 1 | 0 |
| Avolition | 3 | 3 | 0 | 0 | 1 | 0 | 2 |
| Dizziness | 2 | 1 | 1 | 0 | 0 | 0 | 2 |
| Migraine | 1 | 1 | 0 | 0 | 0 | 0 | 1 |
| Difficulties with coordination and falling | 1 | 0 | 1 | 0 | 1 | 0 | 0 |
| *Comment.* IG = Intervention group. Participants in the standard condition (IG 1) received feedback after every module, while those in the guidance-on-demand condition (IG2) could reach out to E-Coaches as needed. The basic condition (IG3) involved no additional coaching. Rejected =participant rejected SRT from the beginning, discontinued = participants discontinued SRT prematurely, inconsistent = participants inconsistently adhered to SRT, consistent participants consistently adhered to SRT | | | | | | | |

## Supplement 6: Coping strategies

|  |  | **Intervention groups** | | | **Adherence to SRT** | | |
| --- | --- | --- | --- | --- | --- | --- | --- |
| **Coping strategies** | **Number of participants** | **IG1** | **IG2** | **IG3** | **Discontinued** | **Inconsistent** | **Consistent** |
| Enduring | 6 | 5 | 1 |  | 3 | 0 | 3 |
| Social support | 4 | 2 | 2 |  | 0 | 0 | 4 |
| Going for walks | 4 | 3 | 1 |  | 1 | 1 | 2 |
| Sleeping when needed | 3 | 1 | 2 |  | 2 | 0 | 1 |
| Adjusting the day | 3 | 2 | 0 | 1 | 0 | 0 | 3 |
| Adjustments at occupation/ reducing workload | 2 | 1 | 1 | 0 | 0 | 0 | 2 |
| Taking breaks | 3 | 1 | 1 | 1 | 1 | 0 | 2 |
| Caffeine | 2 | 2 | 0 | 0 | 0 | 0 | 2 |
| Motivation quotes (from intervention) | 2 | 1 | 0 | 1 | 0 | 0 | 2 |
| Occupy oneself | 2 | 1 | 0 | 1 | 1 | 0 | 1 |
| Change room | 2 | 2 | 0 | 0 | 0 | 0 | 2 |
| Calling in sick | 1 | 1 | 0 | 0 | 1 | 0 | 0 |
| *Comment.* IG = Intervention group. Participants in the standard condition (IG 1) received feedback after every module, while those in the guidance-on-demand condition (IG2) could reach out to E-Coaches as needed. The basic condition (IG3) involved no additional coaching. Rejected =participant rejected SRT from the beginning, discontinued = participants discontinued SRT prematurely, inconsistent = participants inconsistently adhered to SRT, consistent participants consistently adhered to SRT | | | | | | | |
